# African Swine Fever Virus DNA in Soft Ticks, Senegal

**DOI:** 10.3201/eid1312.071022

**Published:** 2007-12

**Authors:** Laurence Vial, Barbara Wieland, Ferran Jori, Eric Etter, Linda Dixon, François Roger

**Affiliations:** * French Agricultural Research Centre for International Development, Montpellier, France; † Royal Veterinary College, Hatfield, United Kingdom; ‡ University of Pretoria, Onderstepoort, South Africa; § Institut Sénégalais de Recherches Agricoles, Dakar, Senegal; ¶ Institute for Animal Health, Pirbright, United Kingdom

**Keywords:** African swine fever, Senegal, epidemiology, endemics, soft ticks, Ornithodoros sonrai, dispatch

## Abstract

African swine fever is a highly contagious disease of pigs in Africa. Although its persistence in Senegal may be caused by asymptomatic carriers involved in the domestic transmission cycle, we demonstrated that the soft tick *Ornithodoros sonrai* can be naturally infected with the causative agent.

African swine fever (ASF) is one of the most severe diseases of pigs in Africa. It is caused by African swine fever virus (ASFV), an *Asfaviridae* virus, and usually results in acute hemorrhagic fever in susceptible animals with mortality rates up to 100% in some herds (*1*). ASF is defined by the World Organization for Animal Health as a highly contagious disease that can spread rapidly and have serious socioeconomic effects in international trade of pigs or pig products and food security. No treatment or vaccine is currently available, and control is essentially based on sanitary measures (*1*).

ASF is endemic in eastern and southern Africa, where ASFV is maintained either in a sylvatic cycle between warthogs (*Phacochoerus aethiopicus*) or bushpigs (*Potamochoerus* spp.) and soft tick vectors of the *Ornithodoros moubata* complex or in a domestic cycle that involves pigs of local breeds with or without tick involvement (*2**–**4*). Long-term persistence of ASFV caused by the presence of the soft tick vector *O*. *erraticus* (*5*) has also been reported in the Iberian Peninsula.

In west Africa, ASFV has been introduced several times since the 1970s in different countries by importing infected pigs or meat. These imports resulted in massive sporadic outbreaks that have been eradicated (*6*). Senegal has had several outbreaks caused by regular reemergence of ASFV since its first description in 1959, which suggests a unique epidemiology that has not been reported in most west African countries infected with ASFV (*6*). The presence of warthogs (*7*) and the soft tick *O*. *sonrai* (*8*) in Senegal suggest a sylvatic cycle of ASF. *O*. *sonrai* is closely related to *O*. *erraticus* and the *O*. *moubata* complex and shares similar vector competence for some pathogens, such as *Borrelia*, which causes human relapsing fever in Africa (*9*). This article reports preliminary results on potential involvement of *O*. *sonrai* in persistence and transmission of ASFV and discusses the role of reservoirs or vectors in control measures.

## The Study

**Figure 1:**
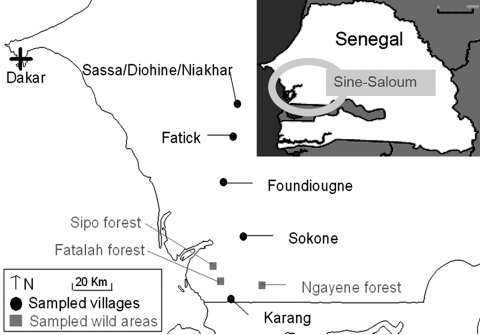
Sampling sites in the Fatick region of Sine-Saloum, Senegal.

Tick investigations were conducted in January 2006 in the Fatick region of Sine-Saloum in west-central Senegal (Figure 1). This region is a major area of pig production and a center for trade with Dakar and Casamance in Sengal and Bissau-Guinea (*10*). Despite no national reporting, ASF outbreaks occur almost every year in Sine-Saloum (*6**,**10*). *O*. *sonrai* has also been found in the Fatick region of Senegal in previous investigations on human relapsing fever (*11*).

**Figure 2:**
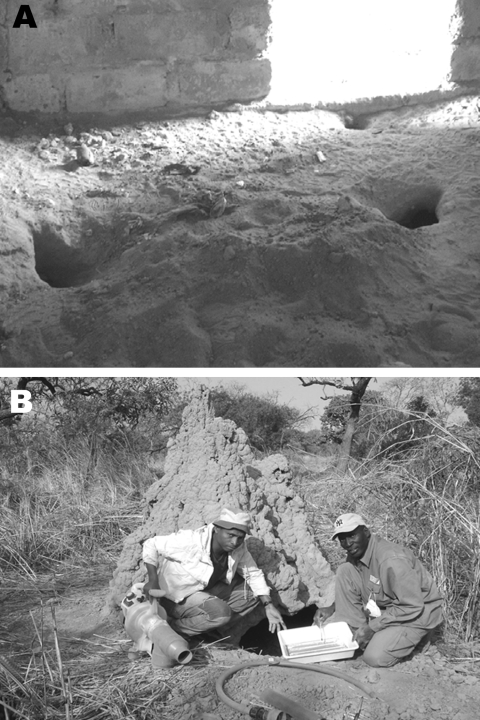
Favorable and unfavorable habitats examined for *Ornithodoros sonrai*. A) Favorable rodent or insectivore burrows infested with *O. sonrai* inside pig buildings. B) Unfavorable warthog burrows negative for *O. sonrai* dug under a termite mound. The portable gasoline-powered vacuum cleaner used for tick collection is also shown.

Three criteria were selected to assess the role of *O*. *sonrai* in ASF (*12*): presence of this tick in domestic pig buildings and warthog habitats, its probability of contact with domestic pigs and warthogs, and its natural infection with ASFV. We searched for *O*. *sonrai* in pigpens in 5 villages or groups of villages, 20–30 km apart per sampling site, along a north-south transect, as well as in warthog burrows in wild areas from 3 different forests (Figure 1). For tick collection, we used a portable gasoline-powered vacuum cleaner adapted for burrow-dwelling ticks (*13*) (Figure 2, panel B). Specimens were stored in liquid nitrogen. Pigpens and warthog burrows were systematically described to determine ecologic preferences of *O*. *sonrai*. Rodent or insectivore burrows, which are known to be favorable natural habitats for *O*. *sonrai*, were also examined at each sampling site to determine the presence or absence of the tick. Collected ticks were tested for ASFV infection by nested PCR amplification of the VP72 gene, a method considered most sensitive for detection of viral DNA in ticks (*14*).

*O*. *sonrai* was found in 11 of the 25 examined pigpens in villages in the 4 most northern sampling sites (Table). Specimens were always found in rodent and insectivore burrows or in deep hollows, in openings inside pig buildings, or near sleeping or foraging areas around pig buildings, as described for the closely related Iberian soft tick *O*. *erraticus* during investigations of ASF (*5*) (Figure 2, panel A). *O*. *sonrai* was not found in litter or buildings, except at 1 farm in Fatick, where nearby burrows were highly infested. The village of Karang showed negative results, even in suitable microhabitats, a finding that confirmed the southern distribution limit of *O*. *sonrai* proposed by Morel (*8*). In wild areas, *O*. *sonrai* was not found in 10 warthog burrows examined (Figure 2, panel B), although its presence was confirmed in contiguous rodent or insectivore burrows. Of 36 ticks tested for ASFV infection, 4 from the 4 most northern sampling sites were positive for ASFV (Table). The farms where ASFV was detected in ticks had reported recent outbreaks in the summers of 2004 and 2005, except for the farm in Fatick. This farm, which belonged to a fattener/collector, had a high turnvover rate of pigs that may have caused difficulties in monitoring their health. Sequencing and BLAST analysis (www.ncbi.nlm.nih.gov) of PCR products confirmed a 100% relationship with ASFV. One sample was positive by repeated analysis. Three samples showed doubtful results when retested by PCR, which indicated low virus titers.

**Table:** Tick collections in villages and detection of African swine fever virus in ticks, Senegal*

Sampling site	Pigpen no.	Burrows	Presence *of Ornithodoros sonrai*†			Infection with *O. sonrai*‡	Last ASF outbreak
			Reference	Within buildings	Near buildings		
Sassa-Niakhar-Diohine	1	11	+	–	+ (4)	0/4	S 2005
	2	6		–	+ (5)	1/6	S 2005
	3	1		–	–		
	4	1		–	–		S 2005
	5	0		–	–		S 2005
Fatick	6	7	+	+ (3)	+ (2)	1/2	
	7	4		+ (2)	+ (2)	0/6	
	8	0		–	–		
	9	1		–	+ (1)	0/1	
	10	2		–	+ (1)		
Foundiougne	11	7	+	–	+ (1)	1/2	S 2005
	12	3		–	–		S 2005
	13	2		–	–		S 2005
	14	1		–	–		Sp 2005
	15	2		–	–		S 2005
Sokone	16	6	+	–	+ (1)	1/7	S 2004
	17	3		–	–		S 2004
	18	1		–	–		
	19	3		–	+ (3)	0/6	
	20	4		–	-		
Karang	21	6	–	–	–		
	22	10		–	–		
	23	2		–	–		S 2005
	24	4		–	–		
	25	2		–	–		

*ASF, African swine fever; +, positive; –, negative; S, summer; Sp, spring. Two other ticks from wild areas in Fatalah and Sipo forests were tested for AFSV and found negative. †Values in parentheses are no. infested burrows or cracks. ‡Values are no. infected ticks/no. tested.

## Conclusions

To our knowledge, this study demonstrated for the first time that *O*. *sonrai* is naturally infected with ASFV. Although these preliminary results suggest a role for *O*. *sonrai* in persistence of ASFV within a sylvatic cycle, only experimental infections will enable formal testing of *O*. *sonrai* as a reservoir and competent vector for AFSV. Additional tick sampling and virus detection analyses are also being conducted to estimate its natural prevalence of infection. If one considers that the ability of pathogens to infect a wide range of hosts is a risk factor for disease reemergence (*15*), our findings are useful for the design of control measures for ASF in Senegal, which currently focus only on pig slaughtering and environment disinfection.

Although contact between soft ticks and domestic pigs has been confirmed in villages in this study, this contact is considered limited. *O*. *sonrai* colonizes mainly rodent or insectivore burrows with high humidity and a cool temperature favorable for its development and survival. It is rare that such favorable microhabitats are near pigpens and enable ticks to feed on pigs instead of small mammals inhabiting burrows. In addition, heterogeneous and relatively low infestation rates for such microhabitats have been shown in a previous study (*11*). Conversely, in wild areas, contact between ticks and warthogs was unlikely, which is contrary to the situation in eastern and southern Africa. In Senegal, warthogs inhabit mainly dry forests and dig superficial burrows under termite mounds, which are not optimal conditions for *O*. *sonrai*. To more clearly quantify contacts between ticks and domestic pigs or warthogs and assess their effect on ASF transmission, analyses of mammalian cytochrome B in tick blood meals and detection of antibodies to tick saliva in serum samples of pigs and warthogs are being conducted.
